# Comparison of vacuum pressures and suction forces generated by different pump systems for aspiration thrombectomy

**DOI:** 10.3389/fneur.2022.978584

**Published:** 2022-10-06

**Authors:** Sum Kim, Jong Young Lee

**Affiliations:** Department of Neurosurgery, Kangdong Sacred Heart Hospital, Hallym University College of Medicine, Seoul, South Korea

**Keywords:** aspiration thrombectomy, vacuum pressure, suction force, endovascular stroke treatment, pump systems

## Abstract

**Objective:**

Aspiration thrombectomy is used to treat endovascular stroke treatment by clot removal through vacuum and suction forces. We aimed to investigate the pressures and suction forces generated by different pump systems for aspiration.

**Methods:**

Vacuum pressure was measured using a vacuum gauge with a closed tip for a 60cc syringe and aspiration pumps. Using an artificial thrombus made from polyvinyl alcohol hydrogel and latex membrane, we assessed the catheter tip force generated on an artificial thrombus using 5Fr Sofia and 6Fr Sofia PLUS intermediate catheters combined with Penumbra Jet Engine or Stryker Medela AXS Universal Aspiration Set. Subsequently, we calculated the catheter tip forces based on the pressure [catheter tip size (force = area × pressure)], and compared with the measured catheter tip force.

**Results:**

The 60cc syringe generated the highest vacuum pressure. Among the automatic pumps, the Penumbra jet engine generated the highest vacuum pressure. The catheter tip forces on the artificial thrombus and latex membrane were 18.5 ± 1.70 and 8.0 ± 1.23 gf, respectively, and 13.9 ± 1.37 and 5.6 ± 0.83 gf, respectively using the 5 Fr Sofia with the Penumbra Jet Engine and the Stryker Medela AXS Universal Aspiration Set, respectively. The corresponding values for the 6 Fr Sofia PLUS with the Penumbra Jet Engine and Stryker Medela AXS Universal Aspiration Set were 39.7 ± 3.88 and 20.7 ± 0.92 gf and 25.4 ± 4.96 and 18.0 ± 0.84 gf. For a constant catheter diameter and the automatic pump, the catheter tip force was significantly larger in the artificial thrombus than latex membrane (*p* < 0.001, ANOVA).

**Conclusion:**

The catheter diameter, vacuum pressure, and clot softness are positively correlated with the catheter tip force.

## Introduction

Since the introduction of intracranial thromboaspiration in 2002, there has been considerable progress in manual aspiration thrombectomy for treating acute ischemic stroke (1–5). Following the success of initial trials on mechanical thrombectomy using stent retrievers to achieve recanalization of occluded large vessels, randomized control trials have shown that frontline aspiration thrombectomy using a direct aspiration at first pass technique is a non-inferior, fast, and effective alternative (6, 7). Accordingly, there has been considerable evolution in aspiration catheter technology. Compared with smaller catheters, larger caliber catheters with greater aspiration forces are associated with shorter procedure times, increased rates of first-pass success, and improved clinical outcomes. Further, aspiration power can be enhanced using automatic aspiration pumps with high aspiration forces, which have started being used by neurointerventional physicians. The 60cc syringe was used before the introduction of automatic pumps (1). There have been inconsistent findings regarding the vacuum pressures generated by a 60cc syringe and automatic pumps according to the study designs (8–10). Additionally, no studies have compared the suction power at the catheter tip when the thrombus is engaged with the catheter. This study aimed to compare the vacuum pressures generated by a 60cc syringe and commercially available automatic pumps as well as the catheter tip forces generated by various combinations of suction catheters and automatic pumps.

## Materials and methods

We evaluated the vacuum pressure generated by different pumps, including the Penumbra Jet Engine (Penumbra, Inc., CA, USA), Penumbra MAX pump, and Stryker Medela AXS Universal Aspiration Set (Stryker, MI, USA). We performed pressure testing of the 60cc syringe to ensure it was comparable to that of the automatic pumps for control. Additionally, we assessed the catheter tip force using 5F Sofia and 6F Sofia Plus catheters (MicroVention, Inc., Tustin, CA, USA) combined with the Penumbra Jet Engine and Stryker Medela AXS Universal Aspiration Set.

### Vacuum pressure

**Figure 1:**
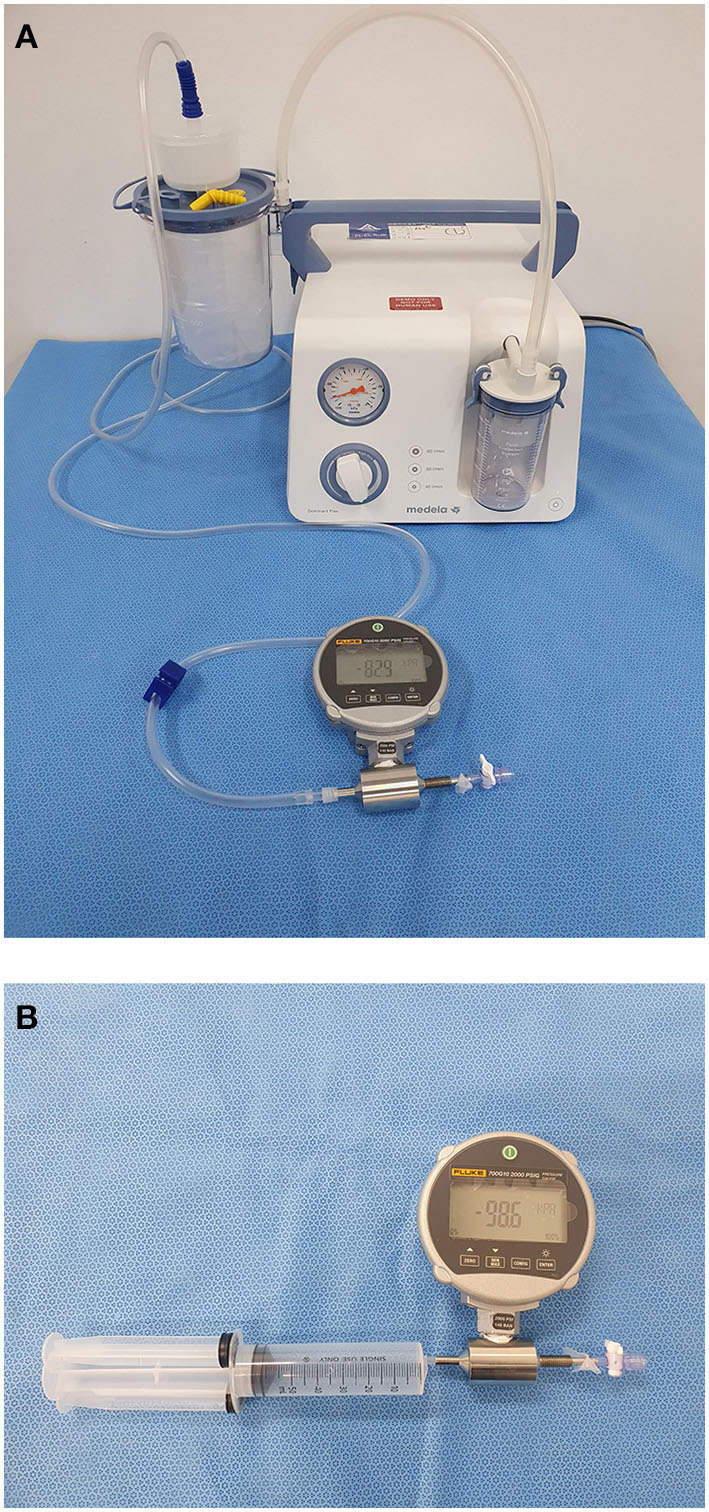
*In vitro* measurement of vacuum pressure. **(A)** The pump was connected to a pressure gauge using a three-way pressure gauge inline adapter (luer lock, ISO 594-2); additionally, the system was completely closed by stopcock, which is attached at the end of the three-way adapter. **(B)** A 60cc syringe was connected to a pressure gauge, and a plunge was locked into place.

We performed *in vitro* analysis of vacuum pressure using a calibrated pressure gauge (Fluke, 700G-10, Everett, WA, USA). The gauge was connected to the pump or 60cc syringe using a three-way pressure gauge inline adapter (Luer lock, ISO 594-2); additionally, a flow stopcock was positioned at the other side of this adapter (Figure 1A). A flow stopcock was completely closed to allow no flow and measure the pure negative pressure generated by the pumps or 60cc syringe at the pre-catheter level. After confirming that the pressure gauge was zero, we turned the pump on and started the timer. To evaluate the trend of the pump-generated negative pressure, we measured the pressure immediately after the pump was turned on, with the pressure trend being observed until the pump was turned off. Subsequently, the pump was turned on and the pressure was measured after 20 min to mimic the actual clinical environment. The flow rate of the Stryker Medela AXS Universal Aspiration Set can be adjusted; moreover, the pressure can be compared by setting the maximum and minimum flow rates. For syringe aspiration, we immediately applied maximal initial pressure, and the plunger was locked into place (Figure 1B). Pressure was recorded in kilopascals (kPa) at a rate of 1 pressure measurement per second.

### Catheter tip force

**Figure 2:**
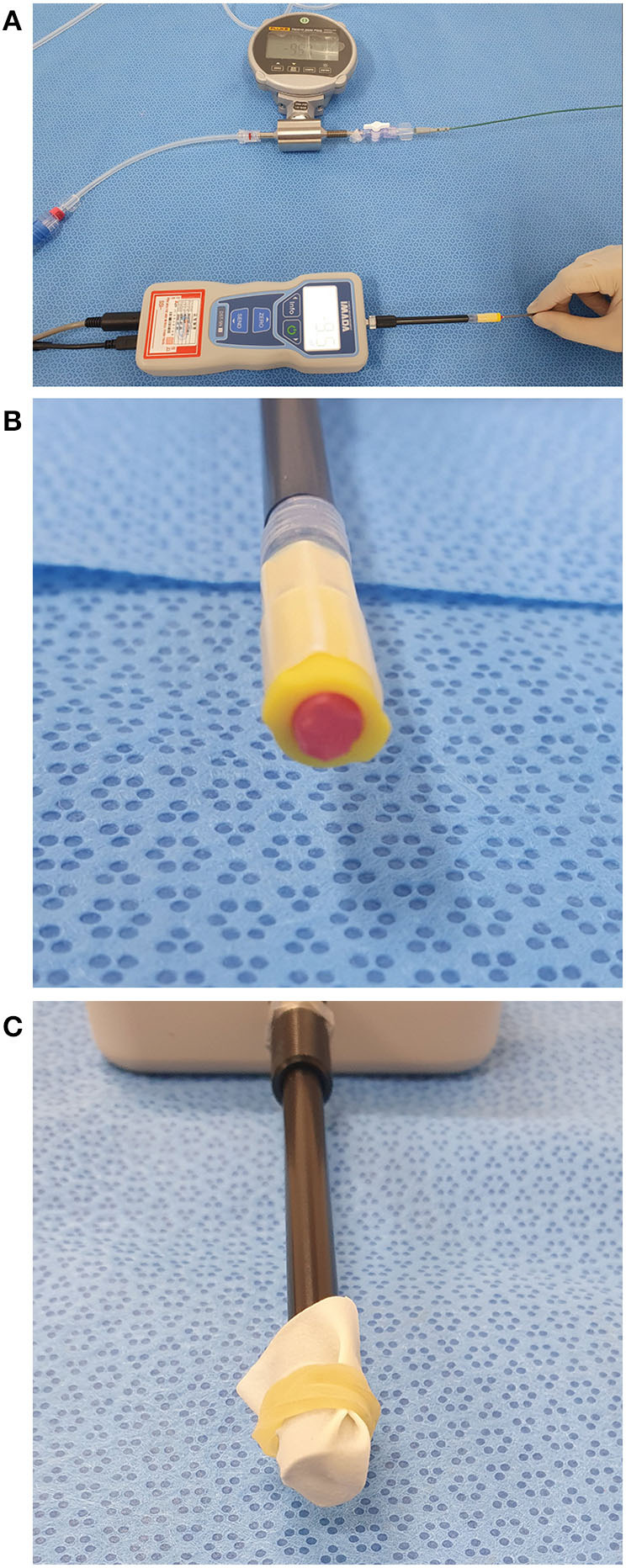
*In vitro* measurement of the catheter tip force. **(A)** A digital force gauge (ZTA-5N, IMADA Co., Toyohashi, Japan) was used to measure the catheter tip force. **(B)** The artificial thrombus was loaded into a 8 mm-sized polypropylene tube and attached at the end of a load cell attached to a digital force gauge. **(C)** A latex membrane was attached at the end of the load cell to simulate hard clots such as white clots.

The catheter tip force was measured using an artificial thrombus made using a polyvinyl alcohol (PVA) polymer and latex membrane. Here, a 10% PVA solution was mixed with sodium tetraborate and sodium bicarbonate. Before experimental use, the artificial thrombus was aged at room temperature for 1 day, loaded into an 8 mm-sized polypropylene tube, and attached at the end of the load cell attached to a digital force gauge (ZTA-5N, IMADA Co., Toyohashi, Japan) (Figures 2A,B). Additionally, to simulate a hard clot, including a white clot, a latex membrane was attached at the end of the load cell instead of the artificial thrombus (Figure 2C). After connecting the Sofia catheters combined with the Penumbra Jet Engine or Stryker Medela AXS Universal Aspiration Set, the pump was turned on and allowed to achieve a steady-state vacuum pressure by remaining on for >10 min. Subsequently, the catheter tip was directly placed onto an artificial thrombus or latex membrane. After confirming good apposition of the catheter tip with the artificial thrombus or latex membrane, the catheter was detached. This procedure was repeated manually 10 times, with continuous measurement of the catheter tip force [gram-force (gf)] throughout the entire procedure. The catheter tip force was defined as the maximum force, which was averaged by selecting the highest values recorded in each session. We compared the measured and calculated catheter tip forces. The catheter tip force was calculated as follows:


$$\begin{array}{l} F_{aspiration}=A_{catheter}\times \Delta P_{catheter}\left(F=AP\right) \end{array}$$

where *F*_aspiration_ is the suction force at the aspiration catheter tip, *A*_catheter_ is the area at the tip, and Δ*P*_catheter_ is the vacuum pressure inside the aspiration catheter. For *A*_*catheter*_, the catheter area was used, while for Δ*P*_*catheter*_, the average value of the measured maximum pressure for each pump was used, as aforementioned.

### Statistics

A one-way analysis of variance (ANOVA) was used to compare the effect of automatic pumps and the 60cc syringe on vacuum pressures and catheter tip forces. Student's *t*-test was used to analyze differences in the vacuum pressures generated by the Stryker Medela AXS Universal Aspiration Set with two different flow rates. All statistical analyses were performed using SPSS 23 software (IBM, Armonk, NY, USA).

## Results

**Figure 3:**
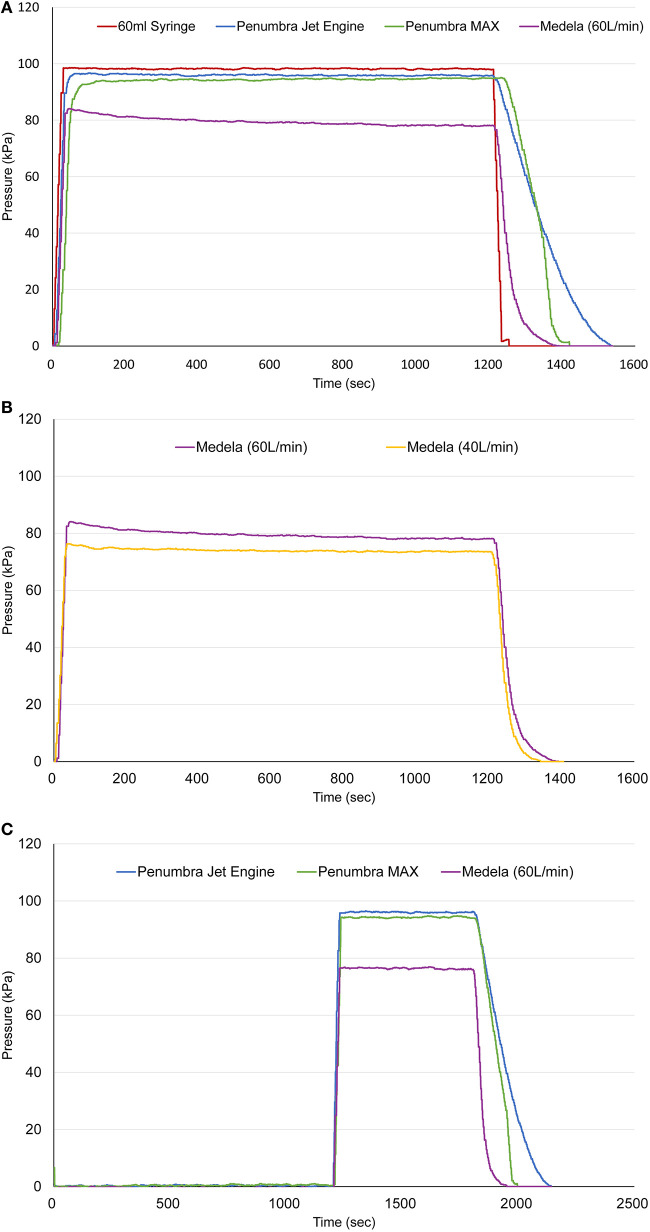
**(A)** Comparison of vacuum pressure between the 60cc syringe and other automatic pumps. **(B)** Vacuum pressure according to flow rate in the Stryker Medela AXS Universal Aspiration Set. **(C)** Vacuum pressures of automatic pumps at 20 min after turning on the pump.

Figure 3A shows the results for vacuum pressure. Compared with the other automatic pumps, the 60cc syringe reached the highest vacuum pressure (average maximum pressure: 98.24 ± 0.16 kPa). The maximum pressures of the Penumbra Jet Engine, Penumbra MAX pump, and Stryker Medela AXS Universal Aspiration Set were 96.01 ± 0.24, 94.31 ± 0.82, and 79.53 ± 1.40 kPa, respectively. There were significant differences in maximum vacuum pressures between the 60cc syringe and the other automatic pumps (*p* < 0.0001). The Stryker Medela AXS Universal Aspiration Set with the maximum flow rate (60 ml/min) reached the maximum pressure (82.91 ± 0.65 kPa) after 35 s. However, the pressure gradually decreased over time and plateaued at 78.77 ± 0.58 kPa after 400 s. After the pump was turned off, when using the 60cc syringe, the pressure reached 0 the fastest, followed by the Stryker Medela AXS Universal Aspiration Set, Penumbra MAX pump, and Penumbra Jet Engine. There was a significant difference in the vacuum pressures of the Stryker Medela AXS Universal Aspiration Set with the two flow rates (Figure 3B). The average maximum pressures with a flow rate of 60 ml/min and 40 ml/min were 79.53 ± 1.40 kPa and 74.06 ± 0.59 kPa, respectively (*p* = 0.001). Under 40 ml/min, the pump reached the maximum pressure (76.23 ± 0.12 kPa) after 35 s, followed by a gradual decrease over time and a plateau at 73.70 ± 0.46 kPa after 400 s. Further, there were significant differences in the maximum pressures of the three automatic pumps at 20 min after turning on the pump (Figure 3C).

**Figure 4:**
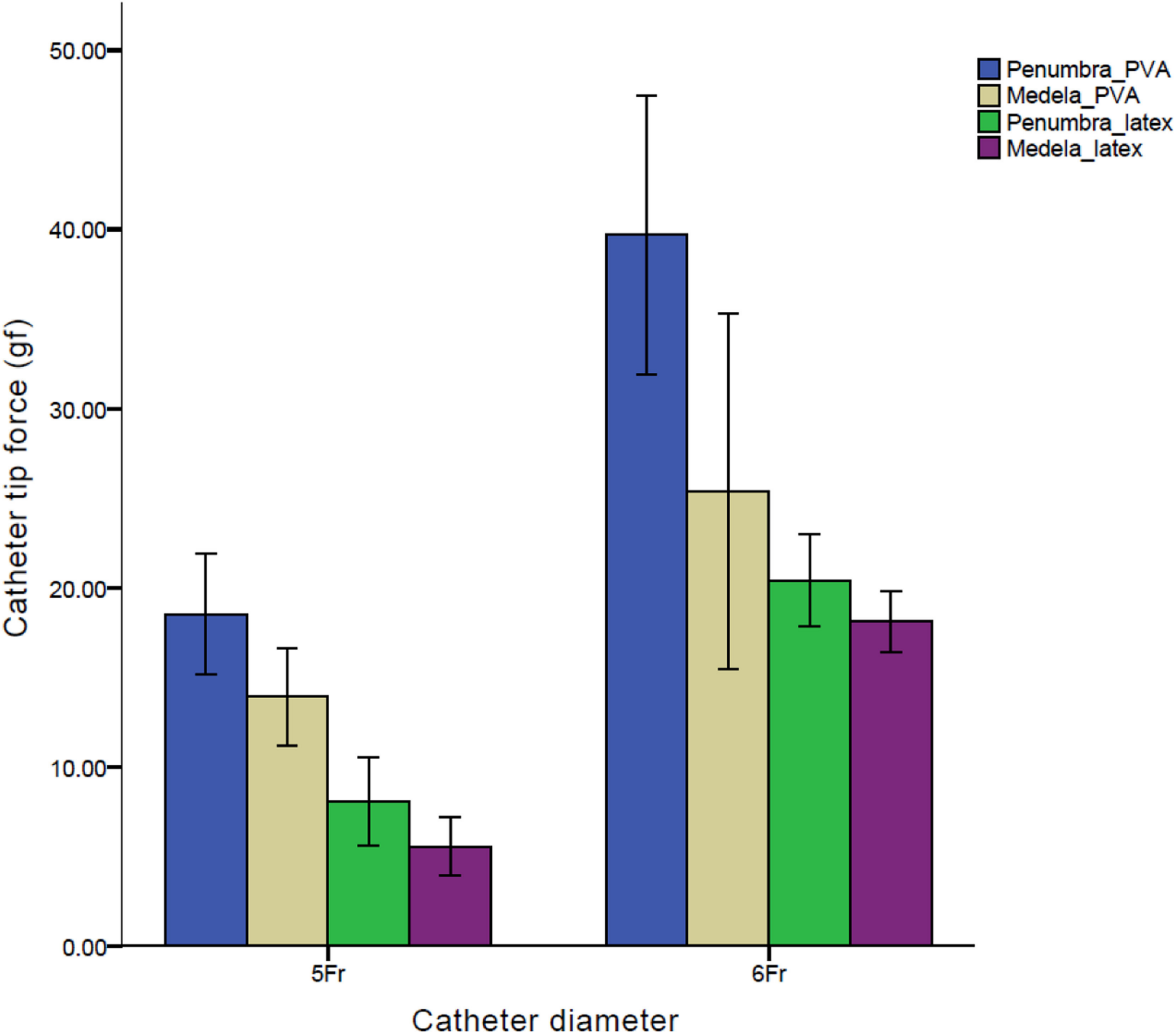
Comparison of catheter tip forces of the 5Fr Sofia and 6Fr Sofia Plus catheters combined with the Penumbra Jet Engine and Stryker Medela AXS Universal Aspiration Set.

In the PVA clot, the catheter tip force was measured to be 18.5 ± 1.70 gf and 39.7 ± 3.88 gf in the 5 Fr Sofia and 6 Fr Sofia PLUS combined with the Penumbra Jet Engine, respectively, as well as 13.9 ± 1.37 and 25.4 ± 4.96 gf in the 5 Fr Sofia and 6 Fr Sofia PLUS combined with the Stryker Medela AXS Universal Aspiration Set, respectively. In the latex membrane, the catheter tip force was measured to be 8.0 ± 1.23 and 20.7 ± 0.92 gf in the 5 Fr Sofia and 6 Fr Sofia PLUS combined with the Penumbra Jet Engine as well as 5.6 ± 0.83 and 18.0 ± 0.84 gf in the 5 Fr Sofia and 6 Fr Sofia PLUS combined with the Stryker Medela AXS Universal Aspiration Set, respectively (Figure 4). Regardless of the mechanical properties of the clot analog, the catheter diameter showed a significant positive correlation with the catheter tip force (*p* < 0.001). For a constant catheter diameter, the catheter tip force was significantly larger in the soft clot (PVA clot analog) than in the hard clot (latex membrane) (*p* < 0.001, ANOVA). The calculated catheter tip force was 12.6 and 22.2 gf in the 5 Fr Sofia and 6 Fr Sofia PLUS combined with the Penumbra Jet Engine, respectively, as well as 10.4 and 18.4 gf in the 5 Fr and 6 Fr Sofia PLUS combined with the Stryker Medela AXS Universal Aspiration Set, respectively. In the PVA clot analog, the measured catheter-tip force was greater than the calculated catheter-tip force. Contrastingly, in the latex membrane, the calculated catheter tip force was greater than the measured catheter tip force.

## Discussion

Mechanical suction thrombectomy involves engaging a thrombus with a large-bore catheter and establishing constant adherence between the thrombus and catheter with suction force. Classically, vacuum forces within a catheter are governed by Poiseuilli's law. Accordingly, studies have used various experimental models to investigate the vacuum flow rate (10, 11). For direct aspiration thrombectomy, the catheter should completely adhere to the thrombus, which causes flow arrest (12). Here, the suction force is directly affected by the applied vacuum pressure and cross-sectional area of the catheter (*F* = *PA*), as well as the diameter and resistance of the vessel wall, the size and mechanical properties of the thrombus, and the blood pressure behind the thrombus (13). However, we could only control the vacuum pressure and catheter diameter during the procedure. Therefore, we compared the vacuum pressure generated by the 60cc syringe and different automatic pumps at the pre-catheter level under a closed system, with the assumption of complete engagement of the thrombus at the aspiration catheter. We found that the 60cc syringe reached the highest vacuum pressure compared with the other automatic pumps. The 60cc syringe achieved a slightly higher vacuum pressure than the Penumbra Jet Engine and Penumbra MAX pumps, which could be because the syringe directly delivers vacuum pressure to the catheter. Conversely, Penumbra pumps deliver vacuum pressure through a silicon tubing set and canister, with a relatively large volumetric space where some pressure might be lost. Further, for the Stryker Medela AXS Universal Aspiration Set, there were differences between the measured and manufacturer-provided maximum pressures, which also significantly differed from the measured maximum pressure of the syringe. Additionally, for the Stryker Medela AXS Universal Aspiration Set, the maximum pressure was rapidly reached, followed by a gradual decrease in the pressure, which eventually plateaued. In addition to the tubing system, we observed a leaking point in ClotFinder^TM^, where the body and lid were not completely sealed. This could have attributed to the drop in pressure and difference in the maximum pressure.

The catheter tip force was measured as the average value of the maximum pressure in different trials, where the catheter was engaged with the clot and pulled until it was removed from the clot. Because the negative pressure could not be continuously maintained using the 60cc syringe, the catheter tip force was compared using automatic pumps. Under the same vacuum pressure, the catheter diameter is positively correlated with the tip force. In addition, when the catheter diameter is constant, a difference in the tip force was confirmed by the difference in the vacuum pressure generated by the automatic pump. Our findings suggest that to successfully perform suction thrombectomy, it is important to use a catheter with a diameter as large as possible in consideration of the target vessel. The vacuum pressure is positively correlated with the tip force; however, it is difficult to suggest an appropriate vacuum pressure since the effective tip force for suction thrombectomy remains unclear. In the first published thromboaspiration, thrombectomy was performed using a 60cc syringe; subsequently, a 60cc syringe was mainly used for suction thrombectomy before the automatic pumps were commercially available (1, 14–16). Additionally, several studies have demonstrated that the 60cc syringe produces the highest maximum pressure and is both safe and cost-effective (14, 17). Therefore, when using an autonomic pump, it is important to use a pump that produces a vacuum pressure similar to that of a 60cc syringe for large vessel occlusions.

The clot softness was positively correlated with the catheter tip force. Since the suctioned material was sucked into the catheter, it changed to a convex shape in the proximal direction of the catheter. The softer the suction material, the more convex it is. Accordingly, soft material is thought to generate a large catheter tip force since the effective cross-sectional area to which the pressure is applied becomes larger than that in the hard material. In the PVA analog, the measured catheter tip force was larger than the calculated one, which could be attributed to the aforementioned phenomenon. Our findings suggest that the success rate of suction thrombectomy might be higher for soft clots than for hard clots. Future *in vitro* and clinical studies are warranted to confirm these findings.

This study had several limitations. First, we used PVA to fabricate a clot analog. Although it served as a good thrombus analog and effectively occluded the catheter tip, it does not fully reflect the mechanical properties of actual clots. Second, we investigated a small number of catheters. However, each catheter, for example, 5F Sofia and 6F Sofia Plus catheters, has a diameter that can represent 5Fr and 6Fr series catheters. Moreover, there is no significant difference in the diameter of catheters with the same series across companies. Since the study objective was comparing suction devices and catheters, absolute force measurement is less important than the between-device differences in values. Additionally, we compared two clot analogs with different mechanical properties and confirmed differences in the catheter tip force according to the mechanical properties of the clot. Human trials are warranted to confirm these findings.

## Conclusion

Compared with other autonomic pumps, the 60cc syringe provides the highest vacuum pressure. Among the autonomic pumps, the Penumbra jet engine provides the highest vacuum pressure and catheter tip force. The vacuum pressure, inner catheter diameter, and clot softness are positively correlated with the catheter tip force on aspiration.

## Data availability statement

The original contributions presented in the study are included in the article/supplementary material, further inquiries can be directed to the corresponding author.

## Author contributions

JL contributed to the conception and design of the study. SK and JL conducted experimental study, data analysis, and wrote the first draft of the manuscript. All authors contributed to manuscript revision, read, and approved the submitted version.

## Funding

This work was supported by the Korea Medical Device Development Fund (RS-2020-KD000058) grant funded by the Korea government (the Ministry of Science and ICT, the Ministry of Trade, Industry and Energy, the Ministry of Health and Welfare, the Ministry of Food and Drug Safety).

## Conflict of interest

The authors declare that the research was conducted in the absence of any commercial or financial relationships that could be construed as a potential conflict of interest.

## Publisher's note

All claims expressed in this article are solely those of the authors and do not necessarily represent those of their affiliated organizations, or those of the publisher, the editors and the reviewers. Any product that may be evaluated in this article, or claim that may be made by its manufacturer, is not guaranteed or endorsed by the publisher.
